# Atmospheric Nonthermal Plasma-Treated PBS Inactivates *Escherichia coli* by Oxidative DNA Damage

**DOI:** 10.1371/journal.pone.0139903

**Published:** 2015-10-13

**Authors:** Adam D. Yost, Suresh G. Joshi

**Affiliations:** 1 Center for Surgical Infections and Biofilms, Department of Microbiology and Immunology, Drexel University College of Medicine, Philadelphia, Pennsylvania, United States of America; 2 Drexel University School of Biomedical Engineering, Science and Health Systems, Philadelphia, Pennsylvania, United States of America; University of Tasmania, AUSTRALIA

## Abstract

We recently reported that phosphate-buffered saline (PBS) treated with nonthermal dielectric-barrier discharge plasma (plasma) acquires strong antimicrobial properties, but the mechanisms underlying bacterial inactivation were not known. The goal of this study is to understand the cellular responses of *Escherichia coli* and to investigate the properties of plasma-activated PBS. The plasma-activated PBS induces severe oxidative stress in *E*. *coli* cells and reactive-oxygen species scavengers, α-tocopherol and catalase, protect *E*. *coli* from cell death. Here we show that the response of *E*. *coli* to plasma-activated PBS is regulated by OxyR and SoxyRS regulons, and mediated predominantly through the expression of *katG* that deactivates plasma-generated oxidants. During compensation of *E*. *coli* in the absence of both *katG* and *katE*, *sodA* and *sodB* are significantly overexpressed in samples exposed to plasma-treated PBS. Microarray analysis found that up-regulation of genes involved in DNA repair, and *E*. *coli* expressing *recA*::*lux* fusion was extremely sensitive to the SOS response upon exposure to plasma-treated PBS. The cellular changes include rapid loss of *E*. *coli* membrane potential and membrane integrity, lipid peroxidation, accumulation of 8-hydroxy-deoxyguinosine (8OHdG), and severe oxidative DNA damage; reveal ultimate DNA disintegration, and cell death. Together, these data suggest that plasma-treated PBS contains hydrogen peroxide and superoxide like reactive species or/and their products which lead to oxidative changes to cell components, and are eventually responsible for cell death.

## Introduction

Reactive oxygen species (ROS) are highly bactericidal [1], and are produced by many antimicrobial agents intracellularly when prokaryotic and eukaryotic cells are exposed. ROS are active free radicals, often inter-convertible, and include peroxide, superoxide, and singlet oxygen. Peroxides such as hydrogen peroxide (H_2_O_2_) generate hydroxyl radicals (OH^.^), a highly toxic ROS, through Fenton and Haber-Weiss reactions [2]. ROS can have a range of deleterious effect on both the host and bacterial cells, comprising a loss of membrane potential and membrane integrity, membrane lipid peroxidation, oxidative changes to cellular protein and DNA. They can block DNA replication and transcription by causing single-strand breaks which accumulate in cells [3–5].

Bacteria have evolved mechanisms to combat ROS bursts, and protect themselves by decomposing ROS, using enzymes such as catalase, glutathione peroxidase, superoxide dismutase, and other cellular antioxidants [6]. Conversely, small amounts of ROS can stimulate cells to produce defensive enzymes and proteins that allow them to tolerate relatively high doses of ROS. The most common mechanism of ROS generation is via intracellular single-electron reduction of molecular oxygen leading to superoxide anion radical (O_2_^**. -**^) formation. Superoxide is then dismutated to H_2_O_2_ by superoxide dismutases. Although *Escherichia coli* have many ROS regulatory systems, the two systems regulated by oxyR and soxR are particularly important. Out of 12 early response proteins, 9 proteins belong to the oxyR regulon, suggesting its importance to the overall management of ROS. OxyR deletion mutant of *E*. *coli* are highly sensitive to H_2_O_2_, leading to rapid loss of viability [7]. ROS are produced under a variety of conditions ranging from normal cellular respiration during metabolism to environmental stress and starvation. During induced oxidative stress, bacteria produce and accumulate relatively high amounts of ROS that increase lethality. Some antimicrobial agents and biocides cause increases to cellular ROS and subsequent death of bacterial cells [7, 8].

Nonthermal plasma treatments are increasingly used in disinfection and sterilization, in different forms of applications, such as direct plasma application over a surface or first treating a liquid of interest, and then applying it over surface. Aqueous solutions treated by plasma in atmospheric air contains a complex cocktail of reactive species such as hydrogen peroxide, superoxide, nitric oxide, nitrogen dioxide, nitrite and nitrate, and typically have a low pH (which helps in stabilizing some of these species) [9]. All of these species are physiologically reactive and known to kill bacteria in suspension [10] by exerting a synergistic antimicrobial effect [9, 11]. Recently, we demonstrated that nonthermal plasma (plasma) -treated solutions acquire strong stable antimicrobial activity, and that these solutions have a rapid and broad spectrum activity against multidrug resistant bacterial pathogens. This activity is retained for at least two years at room temperature [12]. The goal of the present study was to extend upon our preliminary findings (11) to understand the mechanisms underlying the bactericidal effect of plasma-treated PBS solution, using *E*. *coli*’s physiological responses as a probe.

## Materials and Methods

### Floating Electrode-DBD Plasma Set up

The schematic and set up was published recently [4, 12, 13], and shown in **S1 Fig**. The discharge gap between the surface of the plasma electrode and PBS solution was fixed at 2mm. Nonthermal plasma treatment of PBS was optimized, and a predetermined treatment time of 75 seconds was used as sub-lethal dose, unless otherwise specified. Plasma generator / power supply was set to the following parameters: frequency, 1.5 kHz; pulse duration, 10 μseconds; total power output, 6.1 Watts. Total energy applied for 75 seconds of treatment was 90.41 Joules. Dielectric-barrier discharge was created by using a custom copper electrode insulated with polyetherimide and a quartz dielectric (3.81 cm^2^ circular electrode with a discharge area of 5.067 cm^2^). A volume of 125μL of PBS was suspended in a hanging drop slide and treated for 75 seconds. Samples were collected and stored until experimental treatment of *E*. *coli*. The term ‘plasma dose’ and ‘plasma treatment time’ of fluid are synonymously here, and both denote an increasing amount of net plasma energy deposited at the site of plasma discharge. Similarly, the term ‘exposure time’, holding time’ and ‘contact time’ with plasma-treated fluid are synonymous, and indicate the duration of *E*. *coli* cell exposure to plasma-treated PBS. Plasma fluid is synonymously used for plasma-treated PBS solution.

### Bacterial Strains, Culture Condition and Chemicals

Strains of *Escherichia coli* K-12 used in this study are listed in **S1 Table**. These strains were donated by Dr. Xilin Zhao (Public Health Research Institute, Department of Molecular Genetics, Rutgers University New Jersey Medical School, Newark, NJ). The mutants of *oxyR* were donated by Dr. Gisela Storz (Section of Environmental Gene Regulation, NICHD, National Institute of Health, Bethesda, MD). Strains were revived as per their laboratory instructions, and grown in Luria-Bertani broth or on agar [14, 15], with or without selection pressure wherever necessary. The cultures were grown overnight, re-inoculated on the following day to obtain log phase, centrifuged to collect cell pellet, washed twice gently with sterile prewarmed PBS, diluted with prewarmed PBS to an OD600 of 0.2 (unless otherwise specified), and 100 μl of suspension was used for each experiment.

### Colony (Viability) Assay with or without Scavengers

*E*. *coli* cell suspension samples were exposed to either plasma-treated or untreated PBS solution (100 ul:100ul), mixed gently, held over time (from 0.5 min through 15 min) at room temperature, and processed for serial dilution in PBS (pH 7.2) for colony count assay as described [4]. The percentage of surviving cells was calculated for each experiment separately, by counting the number of colonies given by plasma-treated sample divided by number of colonies obtained from untreated sample and multiplied by 100. For ROS scavenging experiments, water soluble vitamin E (D-α-tocopherol; Eastman Kodak Chemical Co, Newport, UK) was used as an universal oxidative stress scavenger while purified (external) catalase (catalase from *Aspergillus niger*, Sigma-Aldrich Corp, St. Louis, MO) was selected as a specific hydrogen peroxide scavenger. 100uL of 150mM water soluble vitamin E or 200 units of catalase (in 100uL) in 1x sterile PBS was pre-incubated with bacteria for 15 minutes prior to the exposure with plasma-treated or untreated PBS solution. The colony counting assay was performed to analyze the amount of protection the water soluble vitamin E and catalase offered the wildtype and gene deficient strains after exposure to plasma-treated PBS. The assays were performed in triplicate and with minimum two independent cultures of wildtype and mutant strains, and the results represented as means ± SEM, and plotted as percentage of viable cells against treatment (exposure) time [16] of cells. All plates were observed for minimum 3 days for growth, unless and otherwise stated.

### Hydrogen Peroxide Detection

The amount of hydrogen peroxide retained in each sample was measured by a commercially available Hydrogen Peroxide Detection Kit (National Diagnostics, Atlanta, GA), following their protocol [6]. A working solution of 20mL, combining the two reagents given in the kit, was added to a 96-well plate at 90μL per well. Serial dilutions of the treated PBS were prepared in order to determine its specific hydrogen peroxide concentration, and 10μL of plasma-treated PBS and its dilutions were added in triplicate to each well. The plate was allowed to incubate at room temperature for 30 minutes for the reaction to be completed. Spectrophotometric measurements were taken at 560nm and normalized versus diluted standard positive control (3% hydrogen peroxide). The findings were graphed as H_2_O_2_ in [mM] versus plasma treatment time [12, 16] of PBS.

### Detection of Lipid Peroxidation Product (MDA)

Lipid peroxides are unstable indicators of oxidative stress in microbial cells that decompose and generate complex reactive compounds such as malondialdehyde (MDA), and 4-nydroxynonenal. The thiobarbituric acid-reactive substance (TBARS) assay is well established assay for monitoring lipid peroxidation responses [4]. Detection of MDA was quantified using OxiSelect TBARS Assay Kit (Cell BioLabs, Inc., San Diego, CA), following the manufacturer’s directions, and as described [4]. Following plasma treatment, the suspensions of samples and controls were centrifuged at 12,000 RPMs for 5 minutes. The supernatant was discarded and the remaining pellet was re-suspended in 100μL of sterile water. SDS lysis solution (100μL) was added to each sample and incubated at room temperature for 5 minutes, after which 250μL of TBA reagent was added to each sample or standard. Tubes were then incubated at 95°C for 60 minutes, and thereafter moved to an ice bath for 5 minutes, and centrifuged at 6000 RPM for 15 minutes. 200μL of the supernatant from each tube was aspirated and deposited in a 96 well plate. Spectrophotometric measurements were taken at 532nm. MDA concentrations for each sample were normalized versus an untreated control, and the findings plotted as MDA in [uM] against treatment (exposure) time [16] of cells.

### Membrane Potential

Measurement of bacteria membrane potential following exposure to plasma treated PBS was carried out via BacLight Bacterial Membrane Potential Kit (Invitrogen / Life Technologies, Grand Island, NY), essentially following the protocol of manufacturer. Following plasma treatment, samples and controls were centrifuged at 5000 RPMs for 5 minutes. The supernatant was aspirated and the remaining pellet was re-suspended in 1mL of sterile water. DiOC_3_ (3, 3’-diethyloxacarbocyanide 3-chlorophenylhydrazone) was added to all tubes except one control (to normalize the baseline staining). Flow-cytometry of samples was performed on the Guava EasyCyte Mini, and analyzed using its software. Results were quantified versus the negative control after collection of 5,000 events per sample. The findings are plotted as percentage of depolarized cells versus treatment (exposure) time [16] of the cells.

### DNA Isolation

DNA was isolated from *E*. *coli* (wildtype and mutants) that were treated or untreated with plasma in absence or presence of catalase supplement. DNA was isolated using DNeasy Blood and Tissue kit (Qiagen, Valencia, CA).

### DNA Electrophoresis

Agarose gel electrophoresis was run to investigate the DNA integrity of each sample *E*. *coli*. In brief, the purified DNA (10μL) was mixed with loading dye (Bioline) and the sample loaded with on 1% agarose gel (containing ethidium bromide, 1 μl/mL) and subjected to electrophoresis using Mini slab, submerged electrophoresis apparatus and power pack (BioRad Laboratories, Richmond, CA), and run for 45 min at constant voltage of 100 volts. A 1kb DNA Ladder (Promega Corporation, Madison, WI) was used for molecular marking in a parallel well. The gel was viewed under UV translluminator and photographs obtained using the attached Gel Documentation System and software (Denville Scientific, Denville, PA).

### Detection of 8-OHdG, the Oxidative DNA Damage Marker

Oxidative DNA damage was measured by standard commercially available EpiQuick 8-OHdG DNA Damage Quantification Direct Kit (Epigentek Group Inc., Farmingdale, NY), using supplied standard controls and following the protocol of the manufacturer. The findings are plotted as 8-OHdG (ng) against exposure time of cells.

### Bioluminescence Assay

A recombinant *E*. *coli* K-12 strain DPD2794 containing a *recA*::*luxCDABE* fusion construct was used in this study, and the assay was performed essentially as described [17, 18]. In brief, the strain was revived from stock by culturing with selection pressure of ampicillin, and then two passages given in Luria-Bartani broth containing 100 ug/ml of ampicillin. The culture then was normalized to 0.2 OD at 600 nm, and 100 ul of it was used for the assay. Thus to each microtube containing 100 ul *E*. *coli* cell suspension, 100 ul of untreated PBS or plasma-treated PBS or 0.3% hydrogen peroxide (positive control) was added, mixed gently, and tubes held in dim light for 1min or 5 min or 10 min at 25°C. After centrifugation at 6000 RPM for 10 minutes, the supernatant was discarded and cell pellet collected, washed gently twice with untreated PBS, and resuspended in 200 ul of Luria-Bartani broth. For the reporter assay, this reaction mix was transferred to a Corning® 96 well solid white flat bottom luminescence assay plate (Corning, Tewksbury, MA), and continuously read in microplate reader (Synergy Mx, BioTek) at 490 nm. The graphs were plotted for specific luminescence intensity (SLM; derived by total luminescence divided by OD_600_) against post-exposure time duration (hour). A visible transcriptional response of these fusions in *E*. *coli* is indicator of DNA damage, without the need to perform enzyme assays or to add luciferase substrate exogenously, and this technique is ultrasensitive [18].

### Primer Design

Forward and reverse primers for detection of genes, *oxyR*, *oxyS*, *soxR*, *soxS*, *sodA*, *sodB*, *katE*, and *katG* are shown in **S2 Table**.

### RNA Isolation and Quantitative Real-Time RT-PCR

To 150 μL of control or plasma-treated sample, 300 μL of RNA Protect solution (Qiagen) was added to make a total volume of 450μL. After gently mixing / vortexing for 5–10 seconds, 150μL was transferred to pre-labeled microcentrifuge tube, and the remaining 300 μL was moved to storage at -80°C for later use. The tubes were allowed to incubate at room temperature for 5 minutes, centrifuged for 10 minutes at 5000 RPM, and then RNA isolation was carried out following the protocol of RNeasy Protection Bacteria Mini Kit (Qiagen). The final eluted RNA was suspended in 30 μL of RNase-free water. Following the isolation, RNA was incubated with Turbo DNase (Ambion, Grand Island, NY) to eliminate genomic DNA contamination. Concentrations and the quality of RNA samples were determined using Nanodrop UV-Vis Spectrophotometer (Eppendorf AG, Hamburg, Germany) by following their protocol. The RNA sample was adjusted to 50 ng/ μL, aliquoted in 20μLvolumes, and stored at -80°C until use. 16S rRNA was used as an internal control, and samples normalized. The RT-PCR protocol was carried out using an Eppendorf MaterCycler Pros and EpigradientS (Eppendorf) using the SYBR green Master Mix, forward and reverse primers, and High Capacity cDNA Reverse Transcriptase kit (Applied Biosystems, Foster City, CA) and a 10 μg RNA template. Amplification was performed in a 10 μL final containing 1.25 μL template DNA (10 ng/μL), with denaturation at 95°C for 10 min, followed by 40 cycles consisting of 15 seconds at 95°C and 1 min at 60°C. Raw data was collected via RealPlex4 software and analyzed in Microsoft Excel for comparative fold increase of genes.

### Microarray Analysis

The microarray analysis of wildtype *E*. *coli* was performed using the established protocol and Affymetrix Genechip® *E*.*coli* Genome 2.0 array system (Affymetrix, Santa Clara, CA) as described [6], and the relevant oxidative stress mediating gene responses are discussed here.

### Statistical Analysis

Data sets were analyzed using Microsoft Excel and verified using GraphPad Prism 4 software (San Diego, CA). The *P* values were derived against corresponding untreated conditions, unless and otherwise stated, and a *P* value of <0.05 was considered significant. All experiments were repeated minimum three times unless stated, and data shown are means ± standard error.

## Results

### Titration of *E*. *coli* viability with different plasma-treated PBS treatments

**Fig 1:**
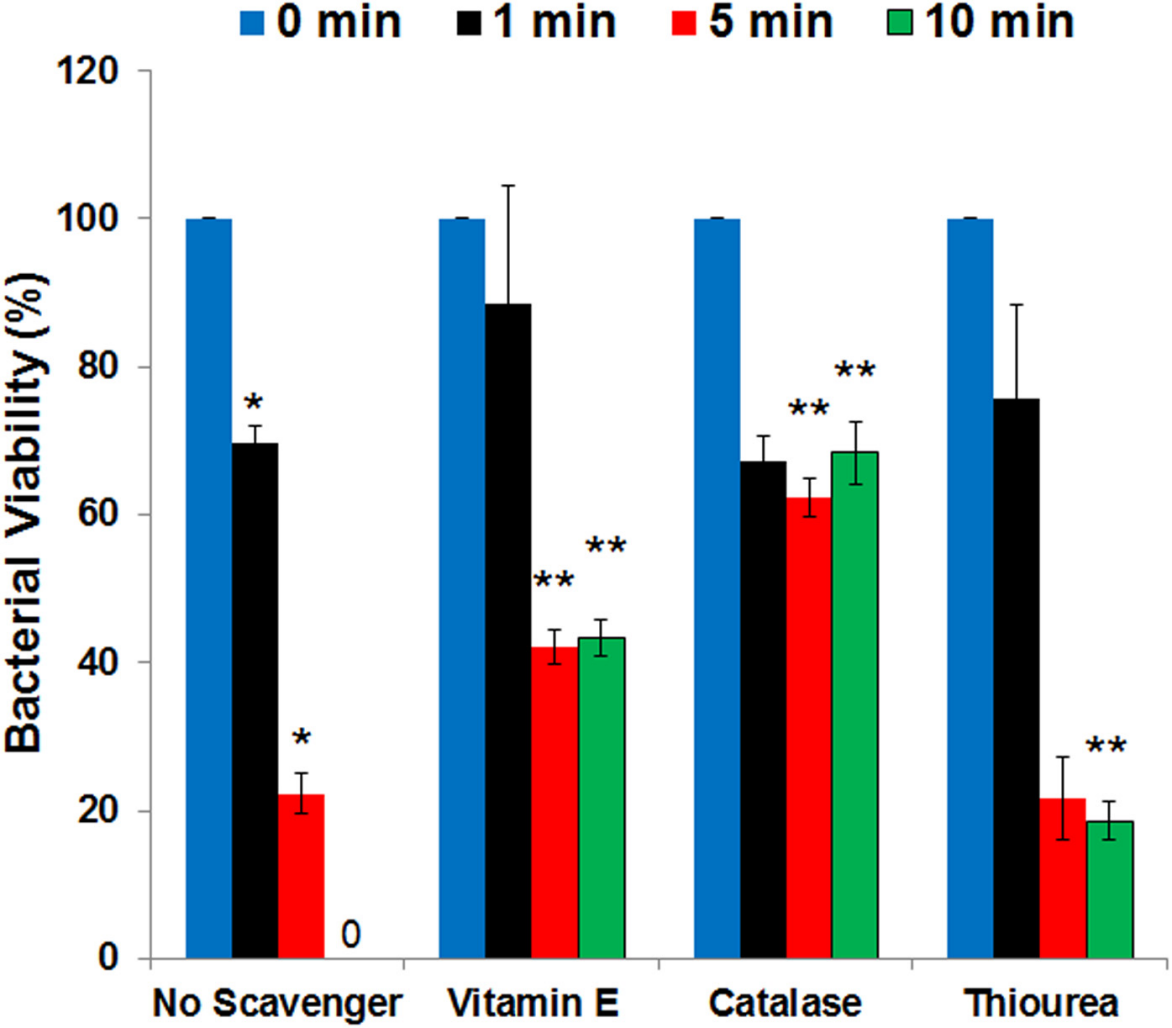
ROS scavengers protect cells under stress conditions. Colony assay demonstrating *E*. *coli* cell viability and the effect of ROS scavengers upon exposure to plasma-treated PBS. Plasma-treated PBS (75 seconds; predetermined) has exhibited a significant, and cell contact (holding) time-dependent bacterial inactivation. A significant protection is provided by preincubation with ROS scavengers (vitamin E, 200mM; calatase, 200 units; thiourea, 100 mM). Bar, SEM; *, *p* <0.05 against plasma untreated (0 min) condition; **, *p* <0.05 against corresponding conditions with ‘No Scavenger’. (n = 3).

The generator of non-thermal DBD plasma used was previously published and shown schematically in **S1 Fig**. In order to best use *E*. *coli* to investigate the properties of plasma-treated PBS, we first optimized the generation of plasma-treated PBS and the treatment of wildtype *E coli* cells over a range of exposure times (holding times or contact times) with a sublethal dose (treated for 75 seconds; pre-determined sublethal dose). *E*. *coli* inactivation after 1 min, 5 min and 10 min of holding is shown in **Fig 1**. The responses are compared with cells that were exposed to untreated PBS for 10 min (shown here as 0 min plasma-fluid exposure time) to calculate relative percentage of cell survival. Colony count assay demonstrated 30% and 80% reduction in cell viability after 1 minute and 5 minutes, respectively. No colonies were observed on plates with samples exposed for 10 min. (**Fig 1**).

### The protective effect of antioxidants

To investigate the oxidative properties of plasma-activated PBS, the protective effects of three antioxidants were tested. Vitamin E (α-tocopherol) was chosen based on its global antioxidant properties; external catalase was chosen to provide specific protection against H_2_O_2_, and thiourea was chosen to scavenge hydroxyl radical-like ROS. Pretreatment of cells with all three scavengers protected *E*. *coli* cells after 1 minute of exposure to plasma-treated PBS, probably indicating a higher activity of ROS-scavenging (Fig 1). A significant increase in cell viability was noticeable after longer holding times, showing a 2-fold increase in cell survival after pre-treatment with α-tocopherol and a 3-fold increase with catalase at 5 minutes. At 10 minute exposure time, wildtype cells were completely inactivated in the absence of a scavenger. The α-tocopherol-protected samples showed ~ 43% viability, while pre-treatment with catalase promoted ~ 72% survival at 10 min of exposure time (**Fig 1**). Thioureaalso protected cells from plasma-activated PBS, allowing ~ 20% cell survival by 10 min stress exposure. Since the peroxide-specific antioxidant provided more effective protection over a global scavenger, we conclude that peroxide such as H_2_O_2_ may be the major intracellular ROS contributing to bacterial inactivation upon exposure to plasma-treated PBS [19–21].

### Physiological damage caused by plasma-treated PBS

**Fig 2:**
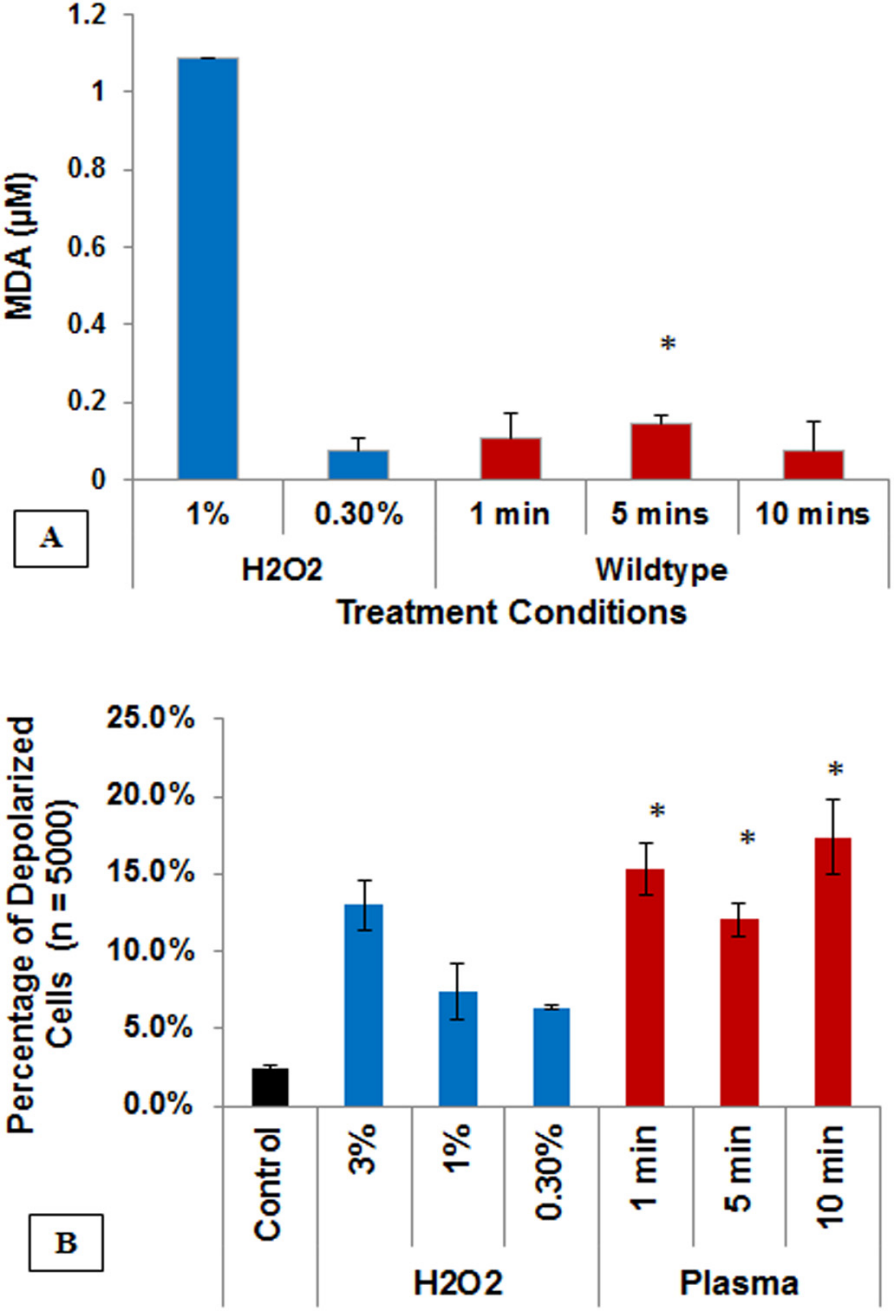
Membrane-associated changes in *E*. *coli* cells (wildtype) following plasma-treated PBS exposure. (A) Malondialdehyde, the marker of lipid peroxidation is detected by TBARS assay demonstrating the peroxidation change in lipid. The findings are normalized against untreated samples. Bar, SEM; *, *p* <0.05 against 0.3% H_2_O_2_. (B) Loss of membrane potential detected by DiOC_3_ assay. Bar, SEM; *, *p* >0.05 against corresponding untreated (control; 0 min) condition. H_2_O_2_ is used as positive control in both assays. (n = 3). The findings together suggest a loss of membrane potential and subsequent membrane lipis peroxidation.

Previously we determined that direct non-thermal plasma treatment of *E*. *coli* causes lipid peroxidation and the formation of malondialdehyde (MDA), a bulky adduct that binds to DNA nucleotides and disrupts DNA replication and repair, leading to cell death [4, 22]. These effects are potentially due to direct physical interaction of plasma-generated charged particles created during pulsed microsecond nonthermal plasma discharge [4, 23]. In order to determine whether bacterial inactivation with plasma-treated PBS also involves membrane dysfunction and lipid peroxidation (instead of direct exposure), we measured the presence of MDA after exposure to plasma-treated PBS. For all holding (contact) time points, we saw appreciable increases in MDA formation [showed 0.11μM (1 min), 0.15μM (5 min), and 0.07μM (10 min)] (after normalizing against untreated samples). Treatment with H_2_O_2_ was used as a positive control (**Fig 2A**), indicating that MDA production with plasma-treated PBS is comparable to 0.3% H_2_O_2_. Thus, plasma-treated PBS at sub-lethal doses also causes lipid peroxidation in *E*. *coli*.

ROS species should either act upon cell membrane directly or by passing through (penetrating) the outer membrane. Therefore, we checked for changes in membrane potential. Membrane potential can affect the transport channels located on the surface, and thus affect permeability and the ROS migration across layer without causing permanent membrane damage [24]. Bacteria exposed to 1 min, 5 min, and 10 min holding times with plasma treated PBS showed respectively, 15%, 12%, and 17% depolarization. These numbers are comparable to depolarization changes measured with 3% H_2_O_2_ (13%) (**Fig 2B**).

### Roles of the SOD and catalase genes in protection from plasma-treated PBS

Next, we looked at specific genes known to protect against oxidation, and at cell survival after treatment. Strains deficient in superoxide dismutase (*sodA* and *sodB*) were selected to test for their role in digestion of superoxides in plasma-treated PBS treatments, and knockout strains of catalase peroxidase (*katE* and *katG*) were chosen to test for the detoxification of hydrogen peroxide-like species.

**Fig 3:**
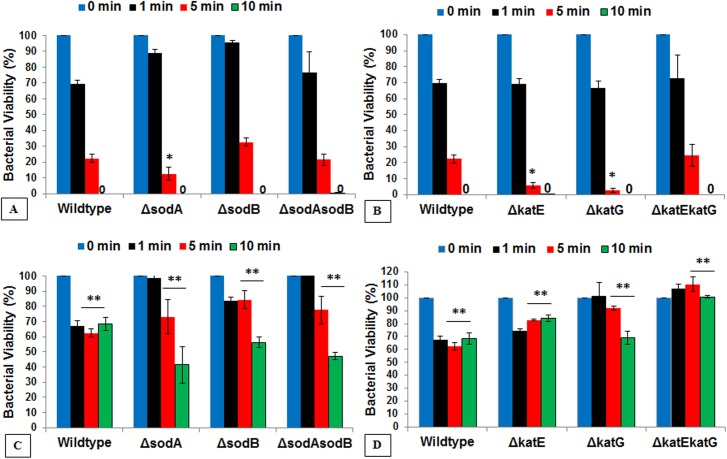
Survival responses of the wildtype and deletion mutants. Colony assays demonstrating viability of *E*. *coli* wildtype and gene deletion mutants of superoxide dismutase (A, C) and catalase (B, D) exposed to dose-dependent plasma-treated PBS without (A, B) or with (C, D) external catalase protection (10 min preincubation with catalase). These findings suggest that external catalase provide a significant protection to wildtype and *sod* and *kat* deletion mutants from oxidative stress. Bar, SEM; *, *p* <0.05 against corresponding condition of wildtype strain; **, *p* <0.05 against corresponding conditions without catalase. (n = 3).

We found that knockout *sodA* was more susceptible to death than wild type or the double mutant *sodA sodB* in general at 1 minute of exposure; and the inactivation was significantly higher after longer exposures to our plasma fluid (5 minutes and 10 minutes) (**Fig 3A**). Surprisingly, the *sodB* superoxide dismutase deficient mutant had slightly higher viability (30%) after 5 minutes of plasma-activated PBS treatment, compared to other strains of ΔsodA (12.5%), ΔsodAsodB (22.3%). In contrast, there were significant differences between wildtype and the catalase-deficient mutants (**Fig 3B**). Unlike the variation in viability found in the single knockouts of the superoxide dismutase strains, both the *ΔkatE* and *ΔkatG* strains were significantly less viable after 5 minutes exposure to plasma fluid than wild type, with only 5.6% and 2.9% survival, respectively. However, the double knockout strain of catalase was indistinguishable from wild type, indicating that other alternative pathways may be involved in causing increased cell death in response to plasma-treated PBS. This phenomenon has also been documented by other groups studying oxidative ROS lethality with other antimicrobial agents [14].

Similar to the initial scavenging experiments, we pre-incubated deficient strains with external catalase [200 units] for 15 minutes prior to the addition of plasma-treated PBS. This shows that external catalase is able to protect wildtype, *ΔsodA* and *ΔsodB* strains at longer exposures (5 min and 10 min) (**Fig 3C**) (*p* >0.05, against corresponding unprotected cells). External catalase also substantially protected the catalase mutants, which exhibited viability similar to wildtype (**Fig 3D**). The mutants *ΔkatE* and *ΔkatEkatG* maintained comparatively higher viability rates than the wildtype at all plasma exposure time points. Strain *ΔkatG* recorded a value similar to that of the wild type during protection, giving respectively, 69.0% and 68.3% survival rate at 10 minutes. Again, we noticed that double knockout of ΔkatEkatG was able to maintain close to 100% viability after all plasma exposure times when protected with external catalase. Overall, all strains showed substantial viability if protected with a specific scavenger of H_2_O_2_. These results have further consolidated our initial hypothesis on the importance of hydrogen peroxide and its role as the primary ROS responsible for cellular inactivation.

### Plasma-treated PBS-induced oxidative stress and DNA damage

**Fig 4:**
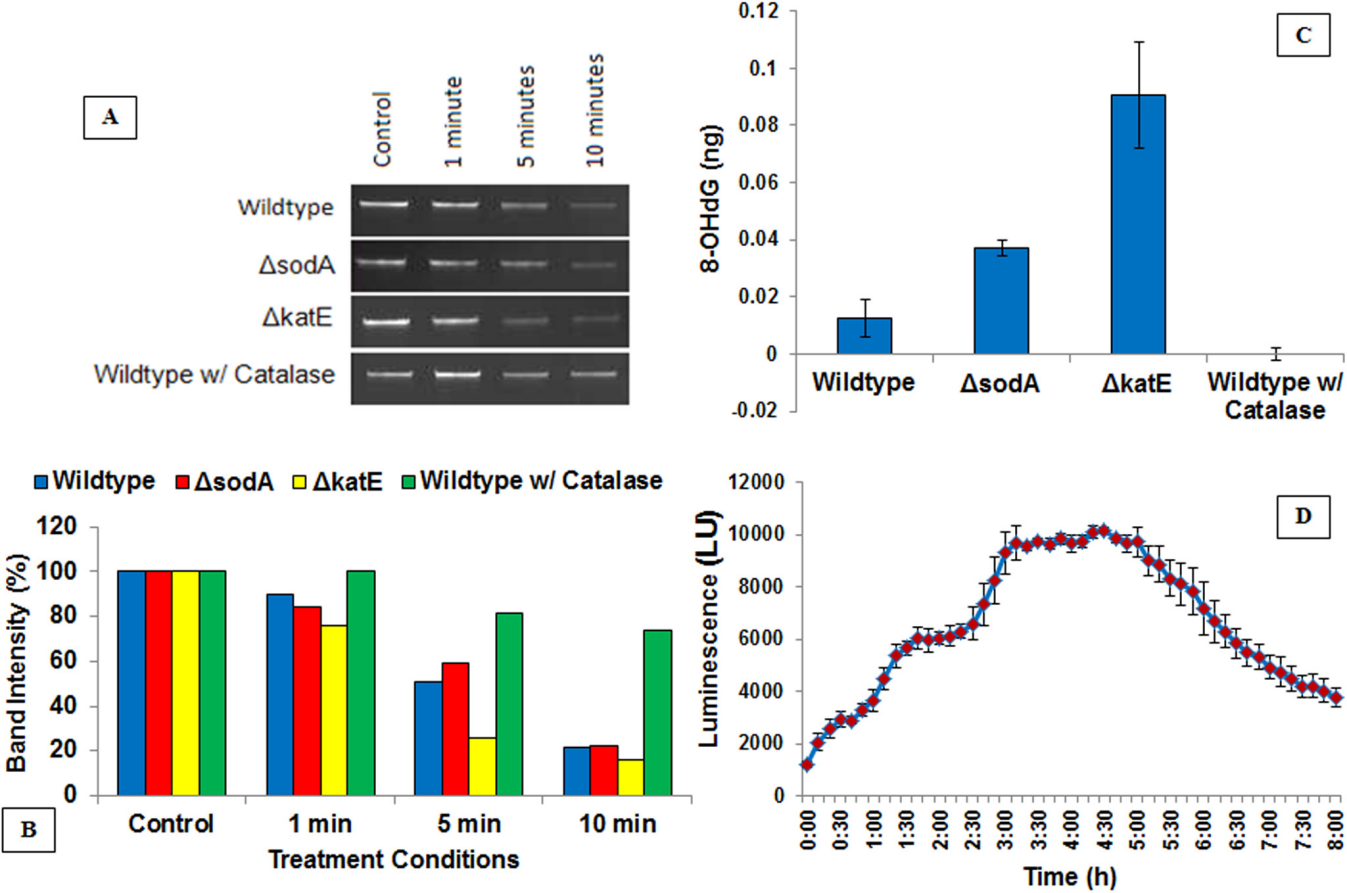
Plasma-treated PBS inducing oxidative DNA damage. (A) A representative agarose gel electrophoresis showing DNA integrity. (B) A graph showing corresponding band intensities of DNA (from gel of Fig 4A). The graph suggests that external catalase substantially protects DNA from damage. (C) The accumulation of 8-hydroxy-2’ -deoxyguanosine (8-OHdG; a marker for oxidative DNA damage) in wildtype and representative deletion mutants after exposure to plasma-treated PBS for 1 minute. Catalase prevented the formation of 8-OHdG during this exposure. Bar, SEM; *, p <0.05 against wildtype *E*. *coli* (without catalase). (D) Response of *E*. *coli* harboring *recA*::*lux* fusion construct to plasma-activated PBS. A maximum induction of recA is seen between 3 h to 5 h post plasma-activated PBS exposure. Bar, SEM. (n = 3).

Following the significant lack of inactivation in presence of ROS scavengers, next, we explored the extent of damage to the DNA upon incubation with plasma-treated PBS. Analysis of DNA integrity was measured by agarose gel electrophoresis of purified strains, with decreasing intensity of the genomic DNA band indicative of increased DNA damage. Based on earlier data, wildtype, *ΔsodA* and *ΔkatE* were selected for DNA damage study. All three types of cells showed severe DNA disintegration at 5 minutes and 10 minutes (**Fig 4A and 4B**), but ΔkatE was most susceptible. As observed with cell viability, catalase protected cells from DNA damage (Fig 4A and 4B; representative figure, showing gel and band intensity graph).

8-OHdG (oxidized deoxyguanosine) is a marker of oxidative damage of DNA, and its relative amount indicates the extent of damage. At 1 minute exposure, we saw classical trend of 8-OHdG, with a significant difference between the level of 8-OHdG in wildtype (0.014 ng) and in *ΔsodA* and *ΔkatE* mutants (0.038 ng and 0.087 ng, respectively), and a complete inhibition of 8-OHdG in the presence of catalase; suggesting that both SodA and catalase give protection to DNA from oxidative-stress mounted by plasma-treated PBS exposure, and that external catalase prevents DNA damage (**Fig 4C**). However, this trend was not seen after 5 minute onwards. There was negligible concentration of 8-OHdG in all 3 strains at 10 minutes of plasma-fluid exposure, indicating that DNA is probably severely damaged or disintegrated by this time (data not shown).

In parallel follow up experiments, we used a recombinant bioluminescent *E*. *coli* with *recA* promoter fused to *luxCDABE* operon (*recA*::*lux*) [17, 18] to demonstrate the influence of plasma-treated PBS on the SOS response to DNA damage; this response was normalized against untreated cells. The kinetics of plasma-treated PBS dose exposure and real-time measurements of bioluminescence, and the specific luminescence intensities (SPI; luminescence /optical densities of cells) are presented here (**Fig 4D**). The results suggest that exposure to plasma-treated PBS leads to slow induction of the SOS response gene, which remains at peak for a 3 h to 5 h induction period. Higher doses of plasma-treated PBS (more than 90 seconds) are lethal to cells, which develop more DNA damage and DNA toxicity, and hence do not see a pattern of induction, but mostly cell death (as luminescence reaction depends on a functional electron transport system, it only functions in viable cells).

### Differential expression of oxidative scavenger pathways in response to plasma-treated PBS

Understanding the importance of the catalase / peroxidase genes and the increasing susceptibility upon their deletion, increased viability in presence of a hydrogen peroxide-specific scavenger, and the slow degradation of DNA due to oxidative stress upon exposure to plasma-treated PBS, we decided to use by quantitative real time polymerase chain reaction (qPCR) to examine the levels of gene expression and to document the involvement of H_2_O_2_ responsive Oxy regulon that governs their transcription. The two pathways that primarily regulate cellular responses to oxidative stress are oxyRS and soxRS [25]. OxyR activation in the presence of hydrogen peroxide modulates a variety of protective proteins, and KatG is important among them [26]. OxyS, similarly regulated, produces a small untranslated mRNA that is used in the repair of DNA/RNA when oxidative damage is present [27]. SoxR, a Fe-S containing activator, regulates the transcription of soxS [28, 29]. SoxS, controlled by autoregulation, activates about 40 different genes responsible for protection from superoxide and nitric oxide [29], including *sodA*, *sodB*, and *sodC* (encoding respectively, Mn-Sod, Fe-Sod and Zn/Cu -Sod)

**Fig 5:**
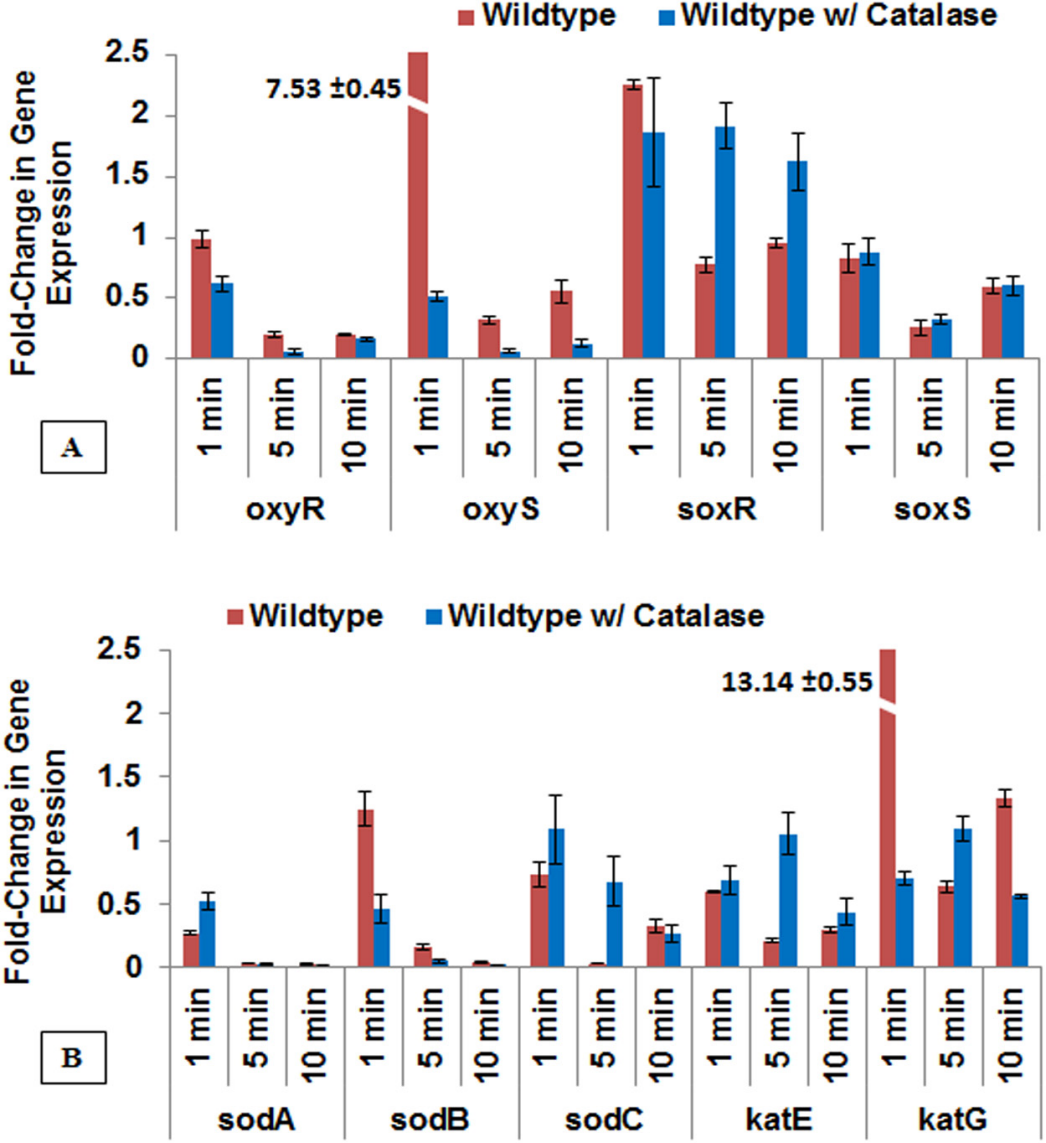
The Graphical presentation showing the change in the levels of gene expression in wildtype *E*. *coli* after plasma-activated PBS treatment and the effect of ROS scavenger, catalase. A differential expression of oxidative stress-responsive oxyRS and soxRS regulons (A), and the regulated genes of superoxide dismutase and catalase (B) are shown. The regulons oxyR, oxyS, soxR and soxS, and the regulated genes *sodB* and *katG* exhibited the early response to exposure to plasma-treated PBS showing activation, and preincubation with external catalase (wildtype w/ catalase) substantially inhibited their activation against longer exposures such as 5 min and 10 min. The data are normalized against corresponding untreated (wildtype without external catalase; no exposure to plasma-treated solution) cells. Bar, SEM. (n = 3).

The genes that showed an increase in expression by 1.5 fold or greater in test samples as compared to untreated controls (0 minute) (after normalization) was considered a significant increase [30]. The wildtype strain produced substantial increases in expression of three genes, *oxyS*, *soxR*, and *katG* after 1 minute of plasma-treated PBS exposure (**Fig 5**). The change in gene expression profiles of *oxyR*, *oxyS soxR* and *soxS* (**Fig 5A**) and *sodA*, *sodB*, *katE* and *katG* (**Fig 5B**) are shown here. The *oxyS* and *katG* produced 7.53 and 13.14 fold changes, respectively, while *soxR* generated a 2.25 fold increase. The expression levels of all genes were measured in the non-significant range for 5 minutes and 10 minutes of exposure. The over-expression of *oxyS* and *katG* in first minute of exposure further indicates the presence of hydrogen peroxide-like species in plasma-treated PBS. Interestingly, the over-expression of *soxR* also indicates the presence of other ROS radicals. While superoxide and nitric acid may be present and generating a measureable response, it is not clear whether these species are a direct result of plasma treatment or H_2_O_2_-responsive changes in the wildtype.

Looking at the high level of induced oxidative stress response and our previous bacterial viability findings, we hypothesize that the addition of external catalase will effectively prevent the activation of selected oxidative stress regulons and defense genes. We found that external catalase was able to successfully repress the over-expression of *oxyS* and *katG* during treatment of wildtype cells (**Fig 5**). However, the expression of *soxR* remained constantly over-expressed for all 3 plasma-fluid holding times (1 min, 1.86 fold; 5 min, 1.91 fold; 10 min, 1.62 fold). We speculate here that overprotection against hydrogen peroxide formation could be placing more stress on alternative ROS defense pathways.

**Fig 6:**
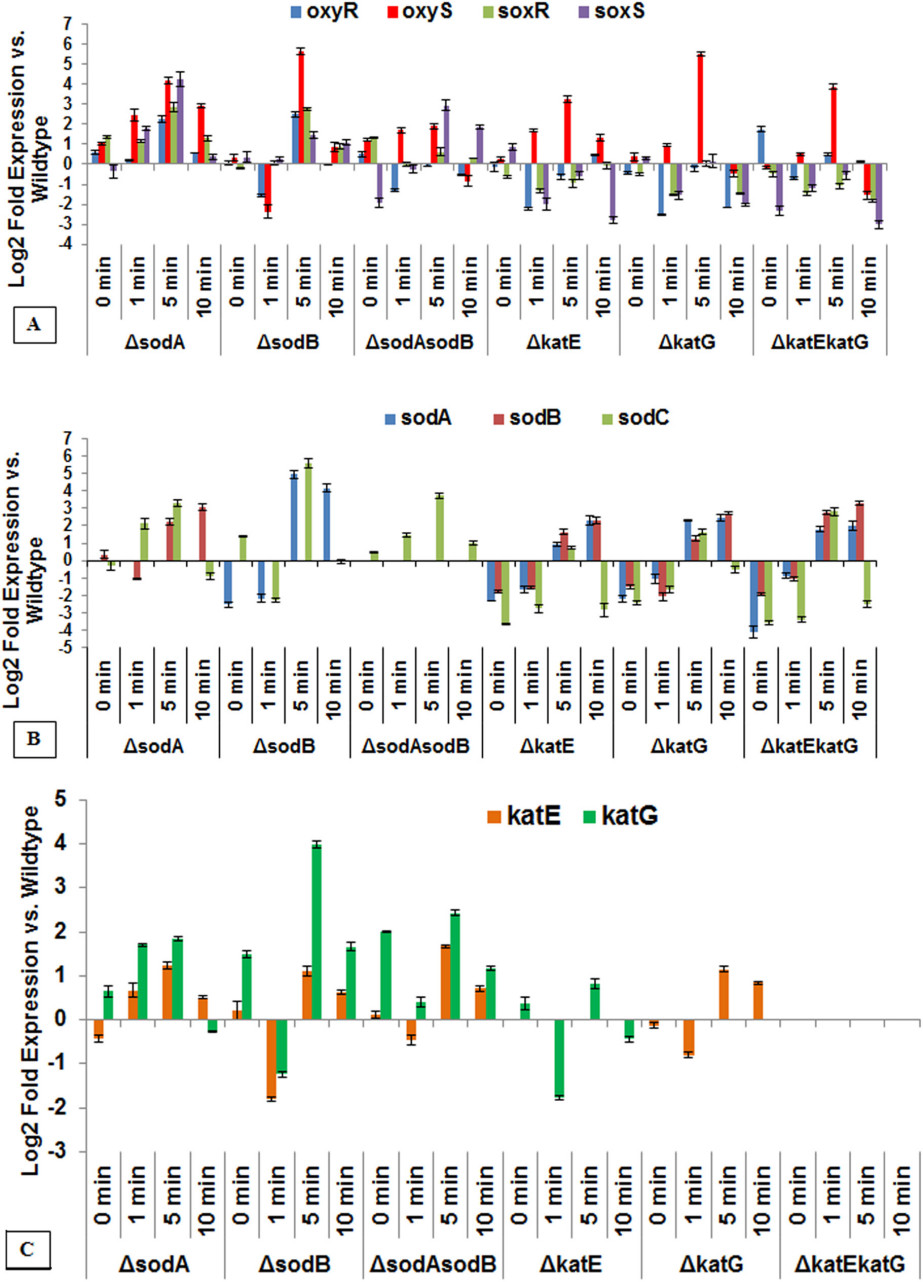
The graphs showing the change in gene expression levels during oxidative stress in gene deficient mutants of *E*. *coli* after plasma treated PBS exposure. The expression is given as fold change against wildtype. The specific RT-PCR probes were used to demonstrate the transcriptional activation response of oxy and sox regulons (A), and the transcription of superoxide dismutase (B) and catalase (C). In all mutants, *oxyS*, *sodC* in *sodA*, *sodB* and their double mutant, and *katG* in *sodA*, *sodB*, *sodAsodB* and *katE* mutants, and *katE* in all *sod* mutants, and *katG* mutant, were activated substantially around 5 min of exposure with plasma-treated PBS. Bar, SEM. (n = 3).

We next sought to determine the effects of plasma-treated PBS on our previously studied oxidative stress defense deficient strains. Expression of the regulons and transcription of genes of interest obtained for all knockouts are compiled and shown in **Fig 6**. The *oxyS* and *katG* genes were significantly expressed in all strains in which they were contained. All deficient strains showed at least a 10 fold increase in *oxyS* expression; the highest fold change (20.12) occurring in *ΔsodA* at 1 minute of plasma fluid exposure (**Fig 6A**). The largest change in *katG* expression (27.37) was also found in the *ΔsodA* strain at the same time point. Interestingly, *katG* was overexpressed at all time-points when *sodB* was knocked out (folds, 1.994 (1 min), 3.591 (5 min), and 1.501 (10 min)) (**Fig 6C**). Both *sodA* and *sodB* were overexpressed at all plasma holding times when neither *katE* nor *katG* were present (**Fig 6B**). However, the same cannot be said about *katE* and *katG* expression in the *ΔsodAsodB* strain. In the latter, instead, we saw a consistently significant expression of *soxS* gene in double mutant. Besides a significant fold increase in the wildtype at 1 minute, the over-expression of *soxR* only occurs with the exclusion of either *sodA* or *sodB*. Finally in the absence of *sodB*, *sodA* was found to be significantly expressed.

In concurrence, we performed microarray assays using the *E*. *coli* wildtype strain to correlate our findings on the involvement of critical genes that mediate oxidative-stress management, and their relative fold expression. A complete list of the major genes which are differentially expressed and showed significantly higher levels of transcription as compared to untreated control were described recently [6]. In addition to the regulons and gene expression response described above (such as *oxyS*, *soxA*, *katG*, *sodA*), we observed the up-regulation of genes involved in glutaredoxin 1 (*grxA*), alkyl hydroperoxidase (*ahpCF*), acinitate hydratase (*acnA*), DNA damage-inducible protein (*dinI*), DNA repair / recombination (*recB*, *recD*, *recN*, *nrdABEFGI*), manganese transporter (*mntH*), and iron-sulfur (Fe-S) cluster assembly proteins (*sufS*, *sufABCDE*).

## Discussion

The antimicrobial property of non-thermal plasma-treated PBS solution was recently reported by our group [4, 12], but the underlying mechanisms of bacterial cell inactivation were not fully described. The production of reactive oxygen species reported by other groups was primarily found using direct plasma jet or plasma afterglow or argon and helium gas plasma [20, 21, 23, 31, 32]. As per our recent observations, plasma-treated solutions retain their strong antibacterial property for about two years. Recently, variable concentrations of singlet oxygen, superoxide, and hydrogen peroxide have been measured in similar (but not identical) systems by different groups [32, 33], and have explained their role as major contributing compounds in plasma treated liquid. Here, we report an entirely different plasma-treated solution and its effect on *E*. *coli* inactivation.

The importance of ROS and their ability to xodize of vital cellular components by antibiotic agents, killing bacterial pathogens has been suspected for a long time, but is only recently being recognized. In many cases, ROS either synergize antibiotic efficacy or mediate bacterial killing [34, 35]. ROS, such as peroxide and superoxide are freely diffusible inside bacterial cells, and once internalized, generate a series of oxidation-reduction reactions mediated by iron-sulfur clusters, flavoproteins, and the Fenton reaction, generating further short-lived but high amounts of hydroxyl radical species [35]. In part this inactivation mechanism involves the oxidation of guanine nucleotide pool [36]. The addition of a general scavenger, α-tocopherol and a peroxide scavenger, catalase, reduced significant *E*. *coli* cell death, whereas thiourea, a scavenger of hydroxyl radicals, inhibited relatively lower percentage of cell inactivation. This may suggest that a lethal amount of hydroxyl radical concentration may not be generated, and that this may not be a major underlying mechanism of inactivation. The α-tocopherol inhibits free radicals-mediated lipid peroxidation of cellular membranes and behaves like a ROS pan-scavenger [4, 37], similar to quinone containing coenzyme. In contrary, catalase is specific and sensitive to H_2_O_2_ concentration, where it converts hydrogen peroxide to water.

Bacteria exposed to plasma treated PBS had moderate membrane lipid peroxidation and changes in membrane potential as compared to hydrogen peroxide control, but these changes were significant as compared to untreated samples. The involvement of lipid peroxidation has been reported during direct plasma treatment of various biological material [4, 38, 39] wherein a compromise in membrane potential, and eventual breach in continuity in membrane is observed. For comparatively low lipid peroxidation, one explanation lies with the overall energy applied to the liquid (smaller dose of plasma energy). Here we applied a sub-lethal dose, capable of stimulating defense pathways to study gene transcription without causing much damage to cellular system under observation. The various ROS contained in the plasma treated liquid could also permeate the membrane without causing much membrane damage [24]. Thus, a change in membrane permeability would be sufficient for these radicals to penetrate the bacterial wall [40]. Additionally, we reported earlier that the equimolar concentration of H_2_O_2_ generated during plasma treatments of fluid is not alone sufficient to completely inactivate *E*. *coli* cells, and a synergy of various ROS is likely occurring [12].

A close correlation exists between bactericidal activity and DNA damage [7, 35, 36]. Therefore, oxidative DNA damage in the presence of ROS pool can be a potential mechanism behind the bactericidal effect of plasma PBS. We saw severe oxidative DNA damage occurring in conjunction with lipid peroxidation. Here we believe that DNA damage could be a double hit phenomenon. Either, the plasma-generated ROS in plasma-treated PBS could have passed through the membrane and oxidized DNA or the formation of lipid peroxidation and their eventual products [2] could have initiated oxidation and subsequent DNA damage, since polyunsaturated side chains of membrane lipids are especially vulnerable to free radical-initiated oxidation. It is believed that the decomposition of hydroperoxides that are mediated by transition metal ion catalysis form more reactive radical species such as hydroxyl radicals, peroxy radicals, and reactive aldehydes (malondialdehyde and 4-hydroxynonenal), leading to more oxidative changes [5, 16]. A substantial concentration of 8-OHdG obtained from our samples indicates oxidative stress of the DNA by base modification rather than strand breaks. Formation of 8-OHdG can often lead to strand misreading, mutagenesis, and cell death [41]. While there is detection of 8-OHdG at 1 minute of exposure to plasma fluid, colony counting data shows minimal change in bacteria viability. This is evident in our wildtype as well as deletion strains. In this early time frame, the DNA repair mechanisms are usually efficient to maintain the DNA functionality and integrity. Over longer exposure times, the levels of oxidized deoxyguanosine slowly decrease due to single and double strain breaks, resulting in total DNA fragmentation. This was confirmed by *recA*::*lux* containing *E*. *coli* bioluminescence. *E*. *coli* strain DPD2794 containing a *recA*::*lux* fusion is very sensitive biosensor for SOS-inducing agents, including hydrogen peroxide [17, 18]. During testing of the wildtype strain, we found that an external catalase supplement could significantly prevent such DNA damage, even upon longer exposure times (5 min and 10 min), indicating that such DNA damage is induced by ROS. A gene *dinI*, encoding DNA damage-inducible protein, and the members of recBCD complex (required in DNA recombination and repair) were also reportedly upregulated during microarray studies [6], further indicating DNA damage. During repeat experiments, we observed a significant amount of 8-OHdG in the wildtype strain at 5 min exposure as compared to deletion mutants of sodA and katE, which could not be justified at this point. The modified base 8OHdG is considered to be one of the oxidative DNA products that are induced by oxygen radicals and is an indicator of DNA damage. It remains to be seen whether *E*. *coli* under plasma fluid-generated ROS experiences both types of DNA strain breaks.

OxyR is a dual transcriptional regulator protein that senses oxidative stress and regulates the antioxidant genes, *katG* (catalase), *ahpC* /*ahpF* (alkyl hydroperoxide reductase), *gorA* (glutathione reductase), and itself (*oxyR*). A hypersensitivity of *oxyR* deletion mutants to H_2_O_2_ demonstrates that it is required to sense H_2_O_2_, and for the subsequent antioxidant gene transcription [1, 42]. In addition, only oxidized OxyR activates these genes to transcription to protect cells from oxidative stress. We have studied OxyRS and SoxRS regulons which are primarily responsible for the activation of bacterial oxidative stress defense genes, and most widely reported redox signaling systems. Previously, we mentioned that *oxyS* is activated when DNA/RNA repair is required under oxidative stress. This in part, supports the findings of the presence of base modifications in DNA of cells treated with plasma treated PBS. Our recent microarray analysis of wildtype strain showed that *oxyS* was most upregulated (29 fold) [6], and RT-PCR analysis also demonstrated a significant upregulation of *oxyS* and *katG* during exposure to plasma-treated PBS. In studies with the wildtype strain, the activation of *oxyR* and the transcription of *oxyS* and *katG* were significantly inhibited in the presence of catalase, indicating the scavenging of hydrogen peroxide-like species. The ahpCF peroxidase and the katG catalase directly limit H_2_O_2_ accumulation [43]. *E*. *coli* has two catalases, HPI (hydroperoxidase I, encoded by *katG*), which functions as a primary scavenger of endogenous H_2_O_2_ and requires a transcriptional regulator, OxyR; and HPII (hydroperoxidase II, encoded by *katE*) which is a key player in survival through stationary phase (via inducible RpoS regulation) and other stresses, and is not induced by H_2_O_2_ [44]. During wildtype *E*. *coli* microarray and wildtype and mutant ΔsodA, ΔsodB and double deletion mutant ΔsodAΔsodB RT-PCR data analysis, we did not see increased transcription of *katE*, wherein *oxyS*, *katG* and *ahpCF* were highly upregulated. This again indicates the involvement of OxyR regulatory control. The transcription factor OxyR also induces expression of *mntH* (encoding a manganese transporter) during H_2_O_2_ stress, which we saw during microarray analysis of the wildtype strain. Manganese import is not required by *E*. *coli* cells to scavenge ROS or to protect DNA from oxidative damage, but is required by the cell to protect enzymes, such as MnSOD (encoded by *sodA*) [45]. The scavenging activity of Mn-containing enzyme exceeds the chemical activity of intracellular concentration of manganese by several orders of magnitude, and therefore Mn is not an efficient scavenger of ROS (as compared to Mn-SOD). The OxyR regulon is also involved in suppression of Fenton reactions (via Dps synthesis) and the oxidation of DNA [45, 46], and in regulation of iron homeostasis through inactivation of Fur protein (required to restore the control of iron import). The Suf system which helps to maintain the activities of Fe-S enzyme cluster is also regulated by OxyR regulon. Most components of the Suf system were found upregulated during microarray analysis of our wildtype strain, but conversely, the proteins involved in iron transport were downregulated (data not shown), and therefore a disruption of iron homeostasis can be expected. Inactivation of Fe-SOD by H_2_O_2_ has been observed earlier [47]. It is reported that just 0.1 uM intracellular H_2_O_2_ is sufficient to activate OxyR regulon to exert its regulatory control in *E*. *coli*. In this context, as per our recent reports [6], and through the present studies, the amount of H_2_O_2_ detected was in the high micromolar range, and was sufficient to generate the required H_2_O_2_ pool inside cells, even upon a brief exposure time of 1 min, and is anticipated to oxidize OxyR.

SoxR transcriptional regulator is responsible for the activation of *soxRS* system, which is also responsive to oxidative stress. In *E*. *coli* the target gene for SoxR is *soxS*, a transcription factor which promotes the expression of several dozen genes that play various roles in oxidative stress management in cells, most notable among them are superoxide dismutases (encodes by *sodA*, *sodB*, *sodC*) and aconitate hydratase (encoded by *acnA*); both extremely sensitive to superoxide accumulation and /or nitric oxide stress. During microarray analysis we saw *sodA* (encoding Mn-SOD) (but not *sodB* and *sodC*) and *acnA* upregulated (both, > 2 folds). Hydrogen peroxide also causes a weak (but appreciable) activation of SodA [48]. There is a significant overlap between the *oxyR* and *soxRS* regulons and other global regulatory networks. For instance, *sodA* gene expression is carried out by four independent control systems [49]. During our wildtype *E*. *coli* studies, we saw that *sodA* (sensitive to superoxide anion) is strongly induced when plasma-treated PBS or 1mM of hydrogen peroxide (control agent) is applied. In contrast, the presence of catalase did not significantly inhibit the activation of *soxR* or transcription of *soxS*, *sodA* and *sodC*. During deletion mutant studies we saw activation of *sodC* in ΔsodA, ΔsodB and ΔsodAsodB, as well as in ΔkatE, ΔkatG, and ΔkatEkatG, indicating a compensatory mechanism of ROS stress management. Thus it is possible that *sodA* transcription is a result of response to both hydrogen peroxide and superoxide, generated by plasma-treated PBS exposure, but may be operated independently of OxyR under the control of SoxRS regulons.

In *E*. *coli*, both Mn-SOD and catalase also mediate acid-stress resistance. Therefore, co-induction of superoxide dismutase and catalase in response to acid accumulation supports the notion that acid-stress and oxidative stress are related phenomena [50]. Plasma treated PBS solution is acidic, and therefore it is possible that this additional acid-stress may be synergizing oxidative stress to activate *oxyR* and *soxRS* regulons. The endogenous SOD levels control iron-dependent hydroxyl radical (HO^.^) formation when cells are exposed to H_2_O_2_, and largely depend on a continuous supply of Fe^2+^. The protection provided by Mn-SOD against H_2_O_2_ is likely by interfering with HO^.^ generation, as we observed the higher transcription of *sodA* (encoding Mn-SOD), and relatively no substantial effect of specific hydroxyl radical inhibitor on survival on wildtype cells. The cytotoxic effects of acid stress and oxidative stress are remarkably similar [50], but a detailed study is required in this direction on plasma fluids.

Recently, SoxR in its reduced form is found to inhibit guanine damage by replacing quinine radicals. Alternatively, SoxR can be activated by the agents that are known to promote DNA damage, such as H_2_O_2_. Indeed, one of the early signals of oxidative stress inside cells is the formation of guanine radical in DNA, which can readily migrate to equilibrate at guanine sites of low oxidation potential, before irreversible oxidation occurs to form 8-OHdG and other base oxidation products [51]. Our observation of the rapid appearance of 8-OHdG and a concurrent activation of soxRS regulon may indicate a redox signaling relation. *soxR* transcription can be activated from a distance through DNA-mediated charge transport *per se* [51]. Altogether, we conclude that hydrogen peroxide is one of the predominant ROS in plasma-treated PBS, and is responsible for bacterial inactivation that activates transcription factor OxyR and SoxR, and leads to oxidative DNA damage in *E*. *coli*.

## Supporting Information

S1 Fig A schematic diagram of non-thermal DBD Plasma treatment set up used in present studies. (PDF)

S1 Table A list of *E*. *coli* derivatives used in present study. (PDF)

S2 Table A list showing *E*. *coli* genes and their corresponding primers used in PCR probing. (PDF)
