# Supplementary figures and images for: Atmospheric Nonthermal Plasma-Treated PBS Inactivates Escherichia coli by Oxidative DNA Damage
Source: PLoS One. 2015 Oct 13;10(10):e0139903. doi: 10.1371/journal.pone.0139903 (PMC4603800; doi:10.1371/journal.pone.0139903)

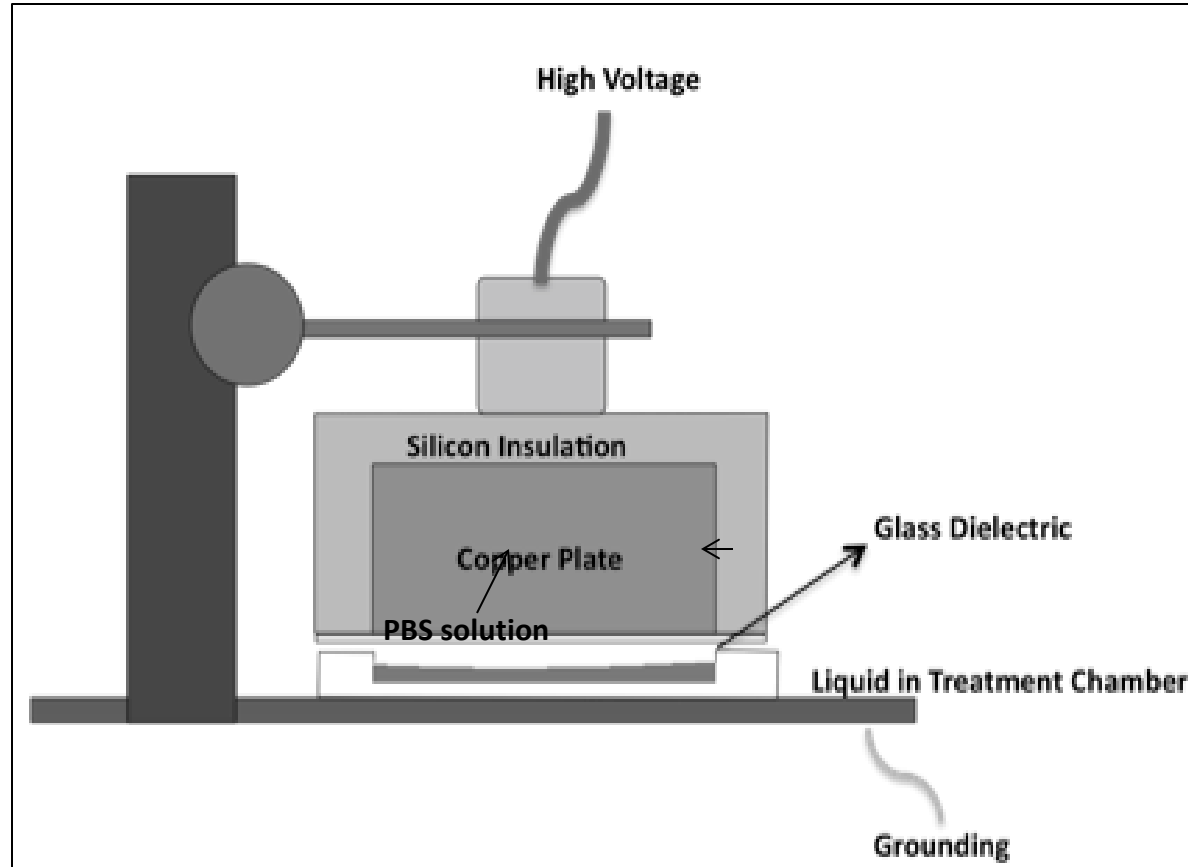

**S1 Fig.** A schematic diagram of non-thermal DBD Plasma treatment set up used in present studies.

Supplement: S1 Fig — (PDF) [file pone.0139903.s001.pdf]
