# Supplementary material for: Atmospheric Nonthermal Plasma-Treated PBS Inactivates Escherichia coli by Oxidative DNA Damage
Source: PLoS One. 2015 Oct 13;10(10):e0139903. doi: 10.1371/journal.pone.0139903 (PMC4603800; doi:10.1371/journal.pone.0139903)
Supplement: S1 Table — (PDF) [file pone.0139903.s002.pdf]

S1 Table. A list of *E. coli* derivatives used in present study.

Strain Number	Relevant Genotype	Source / Reference
BW25113	Wildtype	Xilin Zhao / CGSC#7636
3144	BW25113 ΔsodA	Xilin Zhao
3145	BW25113 ΔsodB	Xilin Zhao
3156	BW25113 ΔsodAΔsodB	Xilin Zhao
3157	BW25113 ΔkatG	Xilin Zhao
3201	BW25113 ΔkatGΔkatE	Xilin Zhao
3202	BW25113 ΔkatE	Xilin Zhao
