# Supplementary material for: Atmospheric Nonthermal Plasma-Treated PBS Inactivates Escherichia coli by Oxidative DNA Damage
Source: PLoS One. 2015 Oct 13;10(10):e0139903. doi: 10.1371/journal.pone.0139903 (PMC4603800; doi:10.1371/journal.pone.0139903)
Supplement: S2 Table — (PDF) [file pone.0139903.s003.pdf]

S2 Table. A list showing *E. coli* genes and their corresponding primers used in PCR probing.

Genes	Accession Number	Forward Primers [5' – 3']	Reverse Primer [5'- 3']
16S rRNA	EG30084	TGCCTGATGGAGGGGGATAA	CCGAAGGTACCCCTCTTTGG
oxyR	EG10681	CCACAGTTGGACCGTACCTG	AACTGGTGGGTCTGTGCTTC
oxyS	EG31116	CCTGGAGATCCGCAAAAGTTC	GCGGCACCTCTTTTAACCCT
soxR	EG10957	GGATCGGCGCATTCATACCT	TTACGCAACGGGCAATCACT
soxS	EG10958	CAAAAATCGGACGCTCGGTG	TCAGACGCTTGGCGATTACA
sodA	EG10953	CCTGCCATCCCTGCCGTATGCTTA	TCGATAGCCGCTTTCAGGTCACCC
sodB	EG10654	AAGATGCTCTGGCACGCCACATTT	TAAACTGCGCTTTGAAATCGGCAA
katE	EG10509	TCCGACTGCCCTTACCATA	GTTCGGTTCGTAATTCGCCG
katG	EG10511	CGATCTACAACCCGACCGAG	ACAGACAGACCAGAATCCGC
